# Prevalence of Nasal colonization with *Staphylococcus aureus* in 4 cities in Peru

**DOI:** 10.1186/s40794-016-0025-x

**Published:** 2016-07-22

**Authors:** Joan Neyra, Michael Ellis, Claudio Rocha, Juan Silvera, Moisés Apolaya, Maruja Bernal, Rina Meza, Enrique Canal, Yocelinda Meza, David Blazes

**Affiliations:** 1 Naval Medical Research Unit N° 6 Lima-Peru (NAMRU-6), 655 Manco Capac St., Miraflores, Lima Peru; 2 University of Toledo College of Medicine and Life Sciences, Toledo, USA; 3 Peruvian Air Force, Lima, Peru; 4 Uniformed Services University (USU), Bethesda, USA

**Keywords:** Antimicrobial resistance, Methicillin-resistant *Staphylococcus aureus*, Military personnel

## Abstract

**Background:**

Antimicrobial resistance (AMR) is a growing public health threat around the world and is not well characterized in the developing setting. Specifically, there is a lack of information regarding nasal colonization with *S. aureus* and methicillin-resistant *Staphylococcus aureus* (MRSA) in Latin America and Peru.

**Methods:**

This is the report of the baseline findings of a prospective cohort study followed up over 1 year at four geographically and ecologically distinct Peruvian Air Force bases in order to determine *S. aureus* nasal colonization prevalence and risk factors. Additionally, all MRSA isolates underwent molecular analysis which included pulsed-field gel electrophoresis and determination of virulence and resistance genes.

**Results:**

We enrolled 756 military personnel. Anterior nares colonization with *Staphylococcus aureus* was detected in 73 of 756 participants (9.7 %) and MRSA was detected in 2 of 756 (0.3 %). Colonization rates differed significantly (*P* = 0.02) between geographic enrollment sites: Talara-4.3 %, Iquitos-9.1 %, Arequipa-14.0 % and Lima-11.3 %. Risk factors for S. aureus colonization included being male and a reported history of respiratory disease.

**Conclusion:**

Overall, we found low prevalence of *S. aureus* and MRSA nasal colonization in this Peruvian military population. These findings contribute to the overall epidemiological understanding of *S. aureus* and MRSA in Latin America. The colonization rates which varied based on geographical location warrants further study.

## Background

The increasing prevalence of methicillin-resistant *Staphylococcus aureus* is a global problem, affecting military and non-military populations around the world. MRSA was first documented in 1960, and until the late 1990s, its presence was confined largely to hospital settings with occasional outbreaks. Since then, the number of outbreaks and infections caused by MRSA, specifically community–associated methicillin-resistant *Staphylococcus aureus* (CA-MRSA) strains increased steadily [1, 2]. USA300 genotype is the predominant CA-MRSA strain recovered from outbreak investigations in U.S. and different countries in Europe and Asia, such as Japan [3], constituting a common cause of community associated skin and soft tissue infections (SSTIs).

Nasal colonization with *Staphylococcus aureus* or MRSA is a risk factor for subsequent infection by these bacteria [4, 5]. In non-Latin American populations, colonization status varies with approximately 20–30 % persistently colonized and 20 % is intermittently colonized [6–8]. Colonization is facilitated by the anatomy of the nasal vestibule and the resistance of *S. aureus* to microbicide peptides in the mucus [9]. Nasal colonization appears to change during one’s lifetime. Colonization begins shortly after birth, decreases during the first 5 years and then it increases until 50 % are carriers between 6 and 12 years; and finally it decreases as children mature and become adults [10]. In the military setting, *Staphylococcus aureus* infections complicate combat-related injuries and produce skin and soft-tissue infections during deployments or training. Among U.S. soldiers, MRSA SSTIs represent a considerable burden, and nasal colonization is a risk factor for subsequent disease [1, 11–14]. For example, the cumulative incidence of SSTIs after 10 weeks of follow-up at training facilities in U.S. military trainees was 38 % in MRSA-colonized subjects while in non-colonized it was only 2 % [11].

The Centers for Disease Control and Prevention’s (CDC) 2013 report on antibiotic resistant pathogens listed MRSA as one of the most serious threats, causing 80,461 severe infections and 11,285 deaths per year in the U.S., leading to a heavy burden of the healthcare system [15]. Similarly, the 2014 WHO Global Health Report on Antimicrobial Resistance reported that in all the WHO regions, MRSA prevalence of infections was above 20 % and increased the risk of death and the associated healthcare costs [16, 17]. In Latin America, a surveillance network for resistant bacterial infections was organized in 1998 under Pan American Health Organization (PAHO) sponsorship; collecting data from specific national and regional hospitals with adequate laboratory infrastructure and resources. However, there is little information regarding the prevalence in other areas, so the geographic extent and the characteristics of MRSA infections in Latin America are not well described in terms of prevalence, isolates, and risk factors [18]. Our current knowledge indicates that four MRSA clones are the most prevalent in Latin America: Brazilian, Pediatric, Cordobes/Chilean and New York/Japan, with marked differences in virulence, antimicrobial resistance profile and geographical distribution [19].

In Peru, current information about MRSA is mostly limited to case reports or series from hospital-based samples from reference hospitals with appropriate protocols for lab procedures and resources. Unfortunately, these studies do not inform us about the prevalence of *S. aureus* nasal colonization nor the epidemiological characteristics in the community, especially in young at-risk populations such as military personnel. Our work was the first study to systematically determine the prevalence and the molecular characteristics of nasal colonization with *Staphylococcus aureus* and MRSA among a Peruvian military population in multiple cities in Peru. Herein, we report the baseline results of this study.

## Methods

### Study design

**Fig. 1:**
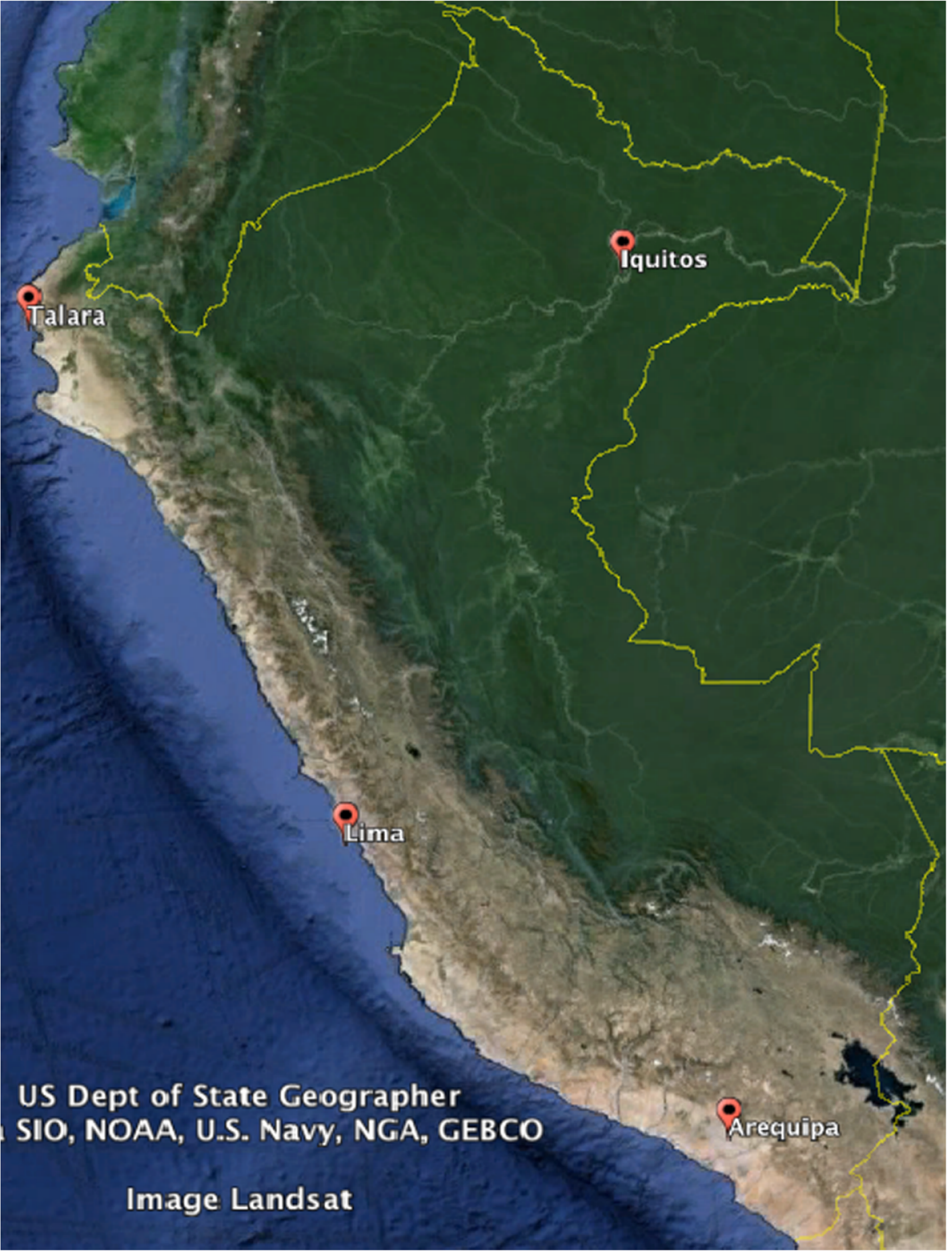
Map of Peru showing the location of the four sites. Lima, the capital city of Peru is located at the central desert coast; Talara, in the northern desert coast; Arequipa, in the southern highlands; and Iquitos is located in the northeastern jungle (Image taken from Google Earth)

We conducted a prospective cohort study with 1 year of follow-up among active duty military personnel from four bases one in each region of the Peruvian Air Force (Lima, Arequipa, Talara, and Iquitos). This is the report of the baseline findings of this cohort. The study population included male and female military active duty personnel, between age 18 and 59 years, stationed at these bases with different climate characteristics (Iquitos is located in the jungle; Talara, in the northern desert coast, Lima at the central coast with mild to warm temperatures depending on the season, and Arequipa, which is located at high altitude in the southern highlands, with a dry and relatively cold climate). See Fig. 1.

### Study procedures

We enrolled military personnel from four large bases in each Air Force region. After we obtained written informed consent, each participant completed a self-administered questionnaire about demographic and risk factors and provided a nasal swab. Investigators obtained a sample from the vestibular area of each of the nares, using BD BBL CultureSwabs® (BD Diagnostic, Sparks MD). Each nasal swab specimen was placed in a refrigerated container and later stored in a refrigerator at 4 °C until the time of the shipping to Naval Medical Research Unit-6 lab (NAMRU-6). Once they arrived at NAMRU-6 Bacteriology lab, the time gap between sample collection and plating was 1 month.

#### Lab procedures

Labeled swabs were shipped to the Naval Medical Research Unit N° 6 (NAMRU-6) located at Lima, Peru. Nasal culture specimens were placed in 5 ml of tryptic soy broth (TSB) supplemented with 6.5 % NaCl and incubated for 18-24 h at 35 °C. After that time, a 75 microL aliquot was plated onto mannitol salt agar. Plates were incubated for up to 48 h at 37 °C and inspected for yellow colonies characteristic of *S. aureus*. Identified isolates were sub cultured onto tryptic soy agar with 5 % sheep blood. Subsequent colonies underwent catalase and coagulase testing per Micro Lab Standard Operational Procedures (SOP) at NAMRU-6. All confirmed *S. aureus* isolates collected from the nasal swabs were shipped to Uniformed Services University (USU)’s Laboratory where they underwent susceptibility testing using disk diffusion tests for identifying MRSA strains, following the standards established by the Clinical and Laboratory Standards Institute [20]. Additionally, all MRSA strains underwent pulsed field gel electrophoresis (PFGE) procedures. Using polymerase chain reaction (PCR) we detected Panton-Valentine Leukocidin (PVL), arginine catabolic mobile element (ACME), staphylococcal chromosome cassete (SCCmec) type, presence of toxic shock syndrome toxin (TST), gene ileS-2 for resistant to mupirocin (mupA), and tolerance to chlorhexidine (qacA/B) using standard protocols.[7].

### Statistical analysis

Overall and baseline colonization status are described based on gender, rank, base of recruitment, age group, time of service in the Peruvian Air Force (≤10 years, 11–20 years, > 20 years), type of activities, smoking status, previous hospitalizations in the last 12 months, previous deployments, use of antibiotics in the last 12 months, previous SSTIs in the last 12 months, previous respiratory diseases, use of corticosteroids in the last 12 months, place of residence, medical conditions; and is reported as numbers and proportions with two-sided 95 % confidence intervals. We also report the prevalence rate of nasal colonization with MRSA strains and the results of the antimicrobial susceptibility for those isolates positive to *Staphylococcus aureus*. We used a logistic regression model to determine the association with the variables listed above. The Institutional Review Boards at USU (Bethesda, MD, USA), and NAMRU-6 (Lima Peru) approved the research protocol.

## Results

### Demographic and clinical characteristics

We enrolled 756 participants. The mean age of the participants was 30.3 ± 11.5 years, but almost 60 % of our study population was younger than 30 years. Slightly more than 80 % were male and 61 % of the participants had more than 10 years of military service. Almost 90 % of the study population included Non-Commissioned officers and troops, and their occupations included administrative (32.5 %), instructional (36.5 % and combat (29.2 %) activities. We found that 20.1 % of the participants had a reported medical condition. Of them, 10 % reported gastrointestinal diseases, 5.4 % reported a skin disease, 2.6 % reported a respiratory disease, and 1.1 % reported an infectious disease. More than a third of the participants (34.8 %) reported the use of antibiotics during the previous year; of these, 52 % (136) gave a specific antibiotic. In addition, 20.2 % reported the use of corticosteroids; and 11.6 % of the enrolled participants were hospitalized during the previous year. Regarding the diagnosis of any SSTI during the previous year, only 5.8 % reported a SSTI. Smoking was common: 34.2 % were current smokers, 16.7 % were past smokers, and 45.6 % reported never smoking (Table 1).

**Table 1:** Demographic characteristics of study participants

Variable	Frequency (*N* = 756)	Percentage (%)
*Age*		
18 – 29 years	450	59.5
30 – 39 years	95	12.6
40 – 49 years	151	20.0
50 years or more	60	7.9
*Time of service*		
10 years or less	463	61.2
11 – 20 years	103	13.6
20 years or more	190	25.1
*Sex*		
Female	146	19.3
Male	610	80.7
*Rank*		
Officers	84	11.1
Non-Commissioned Officers	368	48.7
Troops	304	40.2
*Base of recruitment*		
Iquitos	253	33.5
Arequipa	164	21.7
Talara	162	21.4
Lima	177	23.4
*Administrative activities*		
No	510	67.5
Yes	246	32.5
*Instruction activities*		
No	483	63.9
Yes	273	36.1
*Combat activities*		
No	535	70.8
Yes	221	29.2
*Number of activities*		
Not related	106	14.0
Unique	580	76.7
Multiple	70	9.3
*Place of residence*		
Barracks	250	33.1
Inside the base	114	15.1
Outside the base	392	51.9
*Medical conditions*		
No	604	79.9
Yes	152	20.1
*Number of medical conditions*		
None	604	79.9
One disease	118	15.6
More than one disease	34	4.5
*Use of antibiotics in the previous year*		
No	427	56.5
Yes	263	34.8
Unknown	66	8.7
*Use of corticosteroids in the previous year*		
No	536	70.9
Yes	153	20.2
Unknown	67	8.9
*Hospitalized during the previous year*		
No	645	85.3
Yes	88	11.6
Unknown	23	3.0
*Diagnosis of SSTIs during the previous year*		
No	671	88.8
Yes	44	5.8
Unknown	41	5.4
*Smoking status*		
Never	345	45.6
Past smoker	126	16.7
Current smoker	259	34.2

### Baseline nasal colonization with Staphylococcus aureus and MRSA

The baseline nasal colonization prevalence among the 756 enrolled participants was 9.7 %. There were two periods of recruitment, in October-November 2013 (655 participants) and April-August 2014 (101 participants), and the baseline nasal colonization rates for each period were similar (9.8 % vs. 8.9 %, *p* = 0.7853, Z-statistic for comparison of two proportions). The overall rate of colonization with MRSA was 0.3 % during the study period (2 of 756). These isolates were collected during the 6 month visit at Arequipa, one in a participant enrolled in October 2013 and a second from a different participant who was enrolled in in May 2014). Molecular analysis of these MRSA isolates demonstrated that they possessed SCCmec type IV and qacA/B (chlorhexidine tolerance), but lacked genes for PVL, mupirocin resistance, and toxic shock syndrome toxin (TST).

Table 2 shows statistically significant associations between prevalence of nasal colonization at baseline and demographic and clinical variables. Those participants between 18 and 29 years old and 40 to 49 years old had a higher prevalence of nasal colonization (10.2 % vs. 11.3 %); while, the prevalence was lower than 7 % for other age groups. Nasal colonization was greater in male than female (10.3 vs. 6.8 %, *p* = 0.201). The troops had 11.5 % of nasal baseline nasal colonization, higher than officers or non-commissioned officers (9.5 % vs. 8.2 %). There was a statistically significant difference in the distribution of nasal colonization by base of recruitment (*p* = 0.023); Talara had the lowest baseline prevalence (4.3 %) compared to the other three bases that had similar rates (Iquitos-9.1 %, Arequipa-14.0 % and Lima-11.3 %). Those who lived on base but not in the barracks had the lowest prevalence (5.3 %) but this was not significantly (*p* = 0.198) different from those who lived at the barracks (11.2 %) and those who lived off the base (9.9 %).

**Table 2:** Prevalence of baseline nasal colonization among the different variables under study (*N* = 756)

Variable	Baseline Nasal colonization (%, 95 % CI)		*P* value
	Positive (*n* = 73)	Negative (*n* = 683)	
*Age*			
18 – 29 years	10.2 (7.4 – 13.0)	89.8 (86.9 – 92.6)	0.537^a^
30 – 39 years	6.3 (1.4 – 11.2)	93.7 (88.8 – 98.6)	
40 – 49 years	11.3 (6.2 – 16.3)	88.7 (83.7 – 93.8)	
50 years or more	6.7 (0.3 – 13.0)	93.3 (86.9 – 99.7)	
*Time of service*			
10 years or less	10.6 (7.8 – 13.4)	89.4 (86.6 – 92.2)	0.334
11 – 20 years	5.8 (1.3 – 10.4)	94.2 (89.6 – 98.7)	
20 years or more	9.5 (5.3 – 13.7)	90.5 (86.3 – 94.7)	
*Sex*			
Female	6.8 (2.7 – 10.9)	93.2 (89.0 – 97.3)	0.201
Male	10.3 (7.9 – 12.7)	89.7 (87.3 – 92.1)	
*Rank*			
Officers	9.5 (3.2 – 15.8)	90.5 (84.2 – 96.8)	0.340
Non-Commissioned Officers	8.2 (5.3 – 10.9)	91.8 (89.0 – 94.7)	
Troops	11.5 (7.9 – 15.1)	88.5 (84.9 – 92.1)	
*Base of recruitment*			
Iquitos	9.1 (5.5 – 12.6)	90.9 (87.4 – 94.5)	0.023
Arequipa	14.0 (8.7 – 19.4)	85.9 (80.6 – 91.3)	
Talara	4.3 (11.8 – 7.5)	95.7 (92.5 – 98.8)	
Lima	11.3 (6.6 – 15.9)	88.7 (84.0 – 93.4)	
*Number of activities*			
Not related	10.4 (4.5 – 16.2)	89.6 (83.8 – 95.5)	0.587
Unique	9.1 (6.8 – 11.5)	90.9 (88.5 – 93.2)	
Multiple	12.9 (4.9 – 20.8)	87.1 (79.2 – 95.1)	
*Number of medical conditions*			
None	9.9 (7.5 – 12.3)	90.1 (87.7 – 92.5)	0.122
One disease	6.1 (1.7 – 10.5)	93.9 (89.5 – 98.3)	
More than one disease	17.6 (4.6 – 30.7)	82.4 (69.3 – 95.4)	
*Respiratory diseases the previous year*			
No	9.1 (7.0 – 11.2)	90.9 (88.8 – 92.9)	0.002
Yes	30.0 (9.4 – 50.6)	70.0 (49.4 – 90.6)	
*Use of antibiotics the previous year*			
No	9.1 (6.4 – 11.9)	90.9 (88.1 – 93.6)	0.598
Yes	11.0 (7.2 – 14.8)	88.9 (85.2 – 92.8)	
Unknown	7.6 (11.3 – 14.0)	92.4 (85.9 – 98.9)	
*Use of dicloxacillin previous year*			
No	9.3 (7.2 – 11.4)	90.7 (88.6 – 92.8)	0.005
Yes	33.3 (5.4 – 61.2)	66.7 (38.8 – 94.6)	
*Use of corticosteroids the previous year*			
No	8.9 (6.5 – 11.4)	91.0 (88.6 – 93.5)	0.432
Yes	12.4 (7.2 – 17.7)	87.6 (82.3 – 92.8)	
Unknown	8.9 (2.1 – 15.9)	91.0 (84.1 – 97.9)	
*Hospitalized during the previous year*			
No	9.6 (7.3 – 11.9)	90.4 (88.1 – 92.7)	0.971
Yes	10.2 (3.8 – 16.6)	89.8 (83.4 – 96.2)	
Unknown	8.7 (0.0 – 20.5)	91.2 (79.5 – 100)	
*Diagnosis of SSTIs during the previous year*			
No	9.5 (7.3 – 11.8)	90.5 (88.2 – 92.7)	0.848
Yes	9.1 (0.5 – 17.7)	90.9 (82.3 – 99.5)	
Unknown	12.2 (5.2 – 22.4)	87.8 (77.6 – 97.9)	
*Smoking status*			
Never	8.1 (5.2 – 11.0)	91.9 (88.9 – 94.8)	0.297
Past smoker	12.7 (6.9 – 18.5)	87.3 (81.5 – 93.1)	
Current smoker	10.4 (6.7 – 14.2)	89.6 (85.8 – 93.3)	
*Place of residence*			
Barracks	11.2 (7.3 – 15.1)	88.8 (84.9 – 92.7)	0.198
Inside the base	5.3 (1.1. – 9.4)	94.7 (90.6 – 98.9)	
Outside the base	9.9 (6.9 – 12.9)	90.1 (87.1 – 93.0)	

^a^We used the Fisher’s exact test. For the rest of the variables, we used the Pearson chi square test

Having a respiratory disease increases the prevalence of nasal colonization (30 %) compared with those without respiratory disease (9.1 %, *p* = 0.002). Smoking did not affect the prevalence of nasal colonization; the rates were similar among those who never smoked (8.1 %), previous smokers (12.7 %) and currently smokers (10.4 %) (*p* = 0.300) (Table 3).

**Table 3:** Potential risk factors associated with baseline nasal colonization with *Staphylococcus aureus*

Variable	N	Unadjusted OR	Adjusted OR (95 % CI)	*P*-value
*Use of antibiotics during the previous year*				
No	400	Ref	Ref	
Yes	238	1.2	1.4 (0.8 – 2.6)	0.283
*Hospitalization during the previous year*				
No	566	Ref	Ref	
Yes	72	1.0	1.0 (0.4 – 2.4)	0.980
*Diagnosis of SSTI during the previous year*				
No	600	Ref	Ref	
Yes	38	0.5	0.4 (0.1 – 1.8)	0.237
*Sex*				
Female	122	Ref	Ref	
Male	516	1.8	2.4 (1.0 – 5.7)	0.043
*Base of recruitment*				
Talara	144	Ref	Ref	
Iquitos	207	2.5	2.5 (0.9 – 6.5)	0.065
Lima	149	2.8	2.7 (0.9 – 7.3)	0.051
Arequipa	138	3.9	4.5 (1.7 – 11.9)	0.002
*Smoking status*				
Never smoked	312	Ref	Ref	
Past smoker	107	1.7	1.6 (0.8 – 3.5)	0.204
Current smoker	219	1.2	1.1 (0.6 – 2.1)	0.756
*Respiratory diseases*				
No	619	Ref	Ref	
Yes	19	3.5	4.5 (1.4 – 14.7)	0.014
*Time of service*	638	0.99	0.97 (0.94 – 0.99)	0.030

Among those who used antibiotics during the last year, the nasal colonization prevalence at baseline was not different among those who used them (11.0 %) or not (9.1 %). However, when we analyzed the use of dicloxacillin the previous year, those who reported its use had a 33.3 % prevalence of nasal colonization, compared with those who did not report its use (9.3 %, *p* = 0.005). Regarding the use of corticosteroids, those who used them the previous year, had a prevalence of 12.4 %, which was similar to those who did not use them (8.9 %), (*p*=: 0.432).

In terms of hospitalization during the previous year, the rates were similar among those who were hospitalized (10.2 %) and those who were not (9.6 %) (*p* = 0.971). Similarly there was no difference in prevalence between those who had the diagnosis of SSTIs during the last year (9.1 %) and those who did not (9.5 %). The identified risk factors for S. aureus colonization included being male and a reported history of respiratory disease; while time of service had a slight protective effect.

### Antimicrobial susceptibility of positive isolates and MRSA isolates

NAMRU-6 and USUHS labs processed 183 positive *Staphylococcus aureus* samples from the participants during the study period (1 year). Antimicrobial susceptibility is reported in Table 3. The highest resistance was to erythromycin (16.4 %). In addition, 6.6 % had an inducible resistance to clindamycin. All isolates were susceptible to ceftaroline, trimethoprim-sulfamethoxazole, vancomycin, and linezolid (Table 4).

**Table 4:** Antimicrobial susceptibility of 183 *Staphylococcus aureus* isolates

Antibiotic	Number (%) of samples		
	Susceptible	Resistant	Intermediate
Clindamycin^a^	179 (97.8)	4 (2.2)	-
Erythromycin	153 (83.6)	30 (16.4)	-
Doxycycline	180 (98.4)	-	3 (1.6)
Linezolid	183 (100)	-	-
Oxacillin	181 (98.9)	2 (1.1)	-
Rifampin	183 (100)	-	-
TMP-SMX ^b^	183 (100)	-	-
Vancomycin	183 (100)	-	-
Gentamicin	177 (96.7)	6 (3.3)	-
Levofloxacin	182 (99.5)	1 (0.5)	-
Ceftaroline	183 (100)	-	-

^a^ 6.6 % of the samples showed inducible resistance to clindamycin

^b^TMP-SMX, trimethoprim-sulfamethoxazole

## Discussion

### Baseline prevalence

There are only a few studies of nasal colonization in active duty military populations, primarily from the US and one from China, but none in Peru or Latin America. These studies found that the prevalence of colonization in American recruits was 31 % [11], while the Chinese military found a different rate depending if individuals were deployed to urban (24.6 %) or suburban military centers (16.1 %) [21]. In both cases, the prevalence was higher than what we found at baseline in our study population (9.7 %). Only two previous studies were performed in community settings in Peru. One study included only children from Cajamarca and found a prevalence of 11.9 % [22], while the second included inhabitants of an impoverished community of Lima, and found that among adults of different ages the prevalence of nasal colonization ranged from 20.4 to 39.6 % [23]. These two previous studies in Peru are comparable with others studies performed in Latin American countries that showed that the nasal colonization rates with *Staphylococcus aureus* in community settings was quite varied. In adult populations, the nasal colonization rates in Brazil ranged between 32.7 % [24] and 40.8 % [25]; while among Colombian medical students the rate was 25 % [26]. The prevalence we obtained in the Peruvian military was closer to the prevalence rate reported among adult students in Nigeria (14 %) and healthcare workers in Nicaragua (6.7 to 11.6 %) [27, 28]. In Peru, 3 MRSA strains from Peruvian citizens returning from abroad were characterized in 2011, one was ST30 and the other two were ST8 clones which are related to the USA300 clone [29].

We found differences in the prevalence of nasal colonization based on the site of the geographical site if study enrollment. Of the four sites, Lima is the capital city of Peru (approximately 10 million inhabitants), while Arequipa (900,000 inhabitants) and Iquitos (420,000 inhabitants) are the most important urban centers in the highlands and the jungle and had higher prevalence rates; while Talara, which is a smaller city than the other three (101,000 inhabitants), had only 4.3 %. This colonization prevalence differences may be attributed to a number of factors including climate and ecology, population size, or access to healthcare. An important aspect of population size is that it generally suggests differences in commercial movement, and therefore access to common antibiotics that are still sold without a medical prescription at small pharmacies and drugstores. Additionally, the distribution of antibiotics to each military health facility is based on the most prevalent diseases and the size of the base, which are larger in Lima, Arequipa and Iquitos where administrative and operational regional offices are located, and therefore exceed the population assigned to Talara, which is only an operational base. The increased exposure to antibiotics in Lima, Iquitos and Arequipa might favor the development of nasal colonization with *Staphylococcus aureus* by eliminating other commensal bacteria colonizing the human nares; unfortunately information regarding the most used antibiotics at each of the bases or local areas is currently unavailable.

Dicloxacillin is an antibiotic belonging to the beta lactam family that is used extensively for the treatment of SSTIs. We had expected that the use of dicloxacillin might reduce the rates of nasal colonization with *Staphylococcus aureus*, but we found the opposite. Possibly, the normal microbiota in the anterior nares is more susceptible than *Staphylococcus aureus* to dicloxacillin, therefore favoring the *Staphylococcus aureus* growth. Another possible reason can be the misuse of this antibiotic, which is usually prescribed for 7 to 14 days, but if the dose and time of prescription were not adequate, the antibiotic might have limited effect. We were not able to collect information regarding the dose, time of prescription, where the antibiotics were purchased, or if the treatment was completed.

We identified only two participants with MRSA and therefore our overall prevalence of MRSA colonization during the study period was 0.3 % (2 of 756). These isolates possessed SCCmec type IV which is the characteristic mobile genetic element carrying the mecA gene (methicillin-resistance) found most commonly in CA-MRSA strains. Our MRSA colonization prevalence was close to those reported in previous studies in Peru and Latin America, where it ranges from 0.6 to 1.8 % [23, 30, 31] in communities; however these proportions are lower than those observed in developed countries like US or Europe. In terms of military populations, our results reflect that MRSA nasal colonization is lower than rates reported in US military populations (3 %), while the Chinese study did not detect any MRSA [11, 21].

Our questionnaire was not designed to provide more specific historical information regarding the type of SSTI and treatments prescribed, dose and timing of antibiotics and corticosteroids, time of hospitalization, place, and antibiotics used during this time, which could have given us more detailed information regarding these risk factors. Also, given that this was a self-administered questionnaire, there is the potential for recall bias. In addition, it is possible that some participants did not understand the questions asked or did not know the medical terms used; therefore they left the questions blank. In addition, it is possible that sample handling could have affected the recovery of positive *S. aureus* isolates, specifically during the sampling procedure due to discomfort of the participants that led to movements of the head and a poor quality sample, and the limited available timeframe we had for doing it. Different climate conditions at each site may have affected also the recovery due to the use of a refrigerated container that could not have kept a low temperature at all times.

We believe our results can serve as a proxy to understanding of the nasal colonization with *Staphylococcus aureus* in the community. We found that these isolates have a remarkable antimicrobial susceptibility with very little resistance when compared with other populations. This susceptibility may allow us an optimization of the current treatment of different infections where *Staphylococcus aureus* is a common etiologic agent. This standardization should help to reduce the indiscriminate exposure to more expensive and broader spectrum antibiotics that should be left as second or third line options, which are more expensive and increase the risk of adverse reactions.

## Conclusions

In summary, we found a low prevalence of baseline nasal colonization with *Staphylococcus aureus* (9.7 %) and MRSA (0.3 %) in an active duty military population in Peru. Our results increase the current knowledge about *Staphylococcus aureus* nasal colonization in Peru and Latin America. Further study exploring the geographically differences in *S. aureus* nasal colonization warrants further investigation.

## Abbreviations

AMR, Antimicrobial resistance; MRSA, methicillin-resistant *Staphylococcus aureus*; SCCmec, staphylococcal cassette chromosome mec
